# Association between carpal height ratio and ulnar variance in normal wrist radiography

**DOI:** 10.1186/s12891-024-07647-z

**Published:** 2024-07-09

**Authors:** Anas AR Altamimi, Monther A. Gharaibeh, Muntaser Abu Shokor, Moh’d S. Dawod, Mohammad N. Alswerki, Omar M. Al-Odat, Raghda H. Elkhaldi

**Affiliations:** 1 Orthopedic & Hand Surgery Consultant, Head of special surgery department, Hashemite University, Amman, Jordan; 2 Orthopedic surgery Consultant, Department of Special Surgery, Faculty of Medicine, The Hashemite University, Zarqa, 13133 Jordan; 3 Orthopedic surgery specialist, The specialty hospital, Amman, Jordan; 4 Faculty of Medicine, Mutah University, Al- karak, Jordan; 5 Orthopedic Surgery Resident, Department of Orthopedics, Jordan University Hospital, Amman, Jordan; 6 Orthopedic Surgery Resident, Jordan Hospital, Amman, Jordan; 7 Jordan Red Crescent Hospital, Amman, Jordan; 8 Department of Orthopedic Surgery, Jordan University Hospital, P.O. Box: (13046), Amman, Jordan

**Keywords:** Carpal height ratio, Ulnar Variance, Wrist joint, Distal radius

## Abstract

**Introduction:**

The wrist joint is a complex anatomical structure, and various radiographic parameters are utilized to assess its normal alignment and orientation. Among these parameters are carpal height ratio (CHR) and ulnar variance (UV). Previous literature has indicated that factors such as age and gender may influence these parameters; However, there is a lack of studies investigating these differences specifically in the Middle East or Jordan. Additionally, no prior research has explored the relationship between UV and CHR. Therefore, the objective of this study is to investigate these critical radiological parameters and their associations.

**Methodology:**

A cross-sectional study design was employed, wherein a total of 385 normal wrist X-rays were reviewed, and CHR and UV were measured. Intra-observer and inter-observer reliability assessments were conducted to ensure the consistency and accuracy of measurements. Additionally, the association between UV and CHR was measured and plotted for further analysis.

**Results:**

In our study, the mean CHR was 0.5 (range: 0.4 to 1.5), and the mean UV was − 0.3 mm (range: -5.8 mm to 4.1 mm). We found a significant negative correlation between CHR and age (*p* < 0.05). No significant gender differences were observed in UV and CHR. Additionally, a weak positive correlation was found between UV and CHR (Pearson correlation coefficient = 0.13, *p* = 0.01; adjusted R2 = 0.014, *p* = 0.02).

**Conclusion:**

Age correlated significantly with a decline in carpal height ratio. Additionally, ulnar variance had a week positive yet significant correlation with carpal height ratio.

**Level of evidence:**

Cross-sectional study, Level III.

## Introduction

The wrist joint represents a complex anatomical structure crucial for the proper functioning of the upper extremity, comprising the distal ends of the radius and ulna, the carpal bones, and the proximal regions of the metacarpals [1]. Radiographic evaluation of the wrist involves several key parameters, with the carpal height ratio (CHR) and ulnar variance (UV) being of particular clinical importance [2, 3]. CHR is calculated by measuring the length of the carpal bones height vertically and dividing it by the length of the third metacarpal [4]. UV, the longitudinal distance between the distal articular surfaces of the ulna and radius, is a critical determinant of load transmission through the wrist [5, 6]. These parameters are essential for assessing the biomechanical status of the wrist and identifying potential deviations from normal anatomy.

Pathological alterations in CHR and UV are linked to a range of wrist disorders. A diminished CHR can be indicative of carpal collapse, as seen in advanced arthritis or Kienböck’s disease [7–9], while an increased CHR may suggest carpal instability [10, 11]. UV is equally significant; positive UV is associated with conditions such as ulnar impaction syndrome [12], while negative UV can predispose to Kienböck’s disease due to altered load distribution across the lunate [13, 14]. These radiographic findings are not merely diagnostic markers but also serve as guides for therapeutic interventions, influencing the decision-making process and surgical treatment strategies for wrist pathologies.

CHR and UV can vary across different populations. These variations are influenced by age, gender, ethnicity, and genetics [15–18]. Such diversity in anatomical wrist parameters underscores the importance of population-specific reference standards in orthopedic and radiological assessments. Understanding CHR and UV in Jordanian populations is crucial for tailored diagnosis and treatment of wrist conditions.

Despite the established importance of CHR and UV in radiographic assessments, there is a notable lack of data regarding their normal ranges and interrelations in Middle Eastern populations. Establishing normative data for these parameters among Jordanians is essential, as it will provide a foundation for improved diagnostic accuracy and treatment planning for wrist conditions. Population-specific reference standards are crucial for accurate interpretation of anatomical variations and for ensuring that diagnostic and therapeutic approaches are appropriately tailored to the population being treated.

Therefore, the aim of this study is to investigate the theory that these variations reported in the literature exist in our sample of the Jordanian (Middle Eastern) population and to establish normative reference data for these important parameters. Additionally, this research aims to investigate the association between CHR and UV in a cohort of Jordanian patients with normal wrist radiographs.

## Patients and methods

This study utilized a cross-sectional descriptive design and was conducted at a major teaching hospital in Jordan under the governance of the Hashemite Kingdom of Jordan Ministry of Health. The study spanned a specific period of 6 months (Aug 2020 to Jan 2021). The data collection process involved convenience sampling.

Our study included patients who presented to the orthopedic surgery clinic with non-specific wrist pain during the pre-defined study period and underwent anteroposterior (AP) wrist X-rays. In our study, non-specific wrist pain refers to localized discomfort or soreness in the wrist area that cannot be attributed to a specific cause or identified structural abnormality during initial clinical assessment. This type of pain typically does not correlate with a particular injury, trauma, or observable pathology on routine diagnostic imaging methods such as X-rays or MRIs. Instead, it commonly arises from factors such as repetitive strain, overuse, and minor sprains. The inclusion criteria encompassed all such patients. On the other hand, patients with a history of previous wrist fractures, previous wrist trauma, any previous soft tissue surgeries involving the wrist ligaments, diagnosed inflammatory or non-inflammatory arthritis, and incomplete or improper X-rays were excluded from the study.

In this study, a total of 385 wrist X-rays from 385 patients were evaluated and assessed. Our sample size of 385 participants was determined based on similar previous studies that addressed related topics, as well as practical considerations specific to our hospital’s patient population and data collection capabilities. Additionally, a G power analysis was conducted to ensure statistical adequacy. Using an effect size of 0.5, a significance level (alpha) of 0.05, and a desired power of 0.80, the analysis confirmed that our sample size was sufficient to detect meaningful effects with a high degree of confidence.

All radiographic measurements were conducted by a single orthopedic specialist using the MicroDicom DICOM Viewer, developed by MicroDicom Ltd. in Sofia, Bulgaria. To ensure the reliability of the measurements, a second measurement was performed by the same orthopedic specialist four weeks later to assess intra-observer reliability. Additionally, a third measurement was conducted by another well-trained orthopedic resident to evaluate inter-observer reliability.

An appropriate institutional review board approval was obtained prior to conduction of this study from the Specialty Hospital, Amman, Jordan ethics committee, IRB approval number (102,154/1/5). Appropriate informed consents were obtained from all participants of the study. The Code of Ethics of the World Medical Association (Declaration of Helsinki) was followed while conducting the study.

### Radiographic assessment

**Fig. 1:**
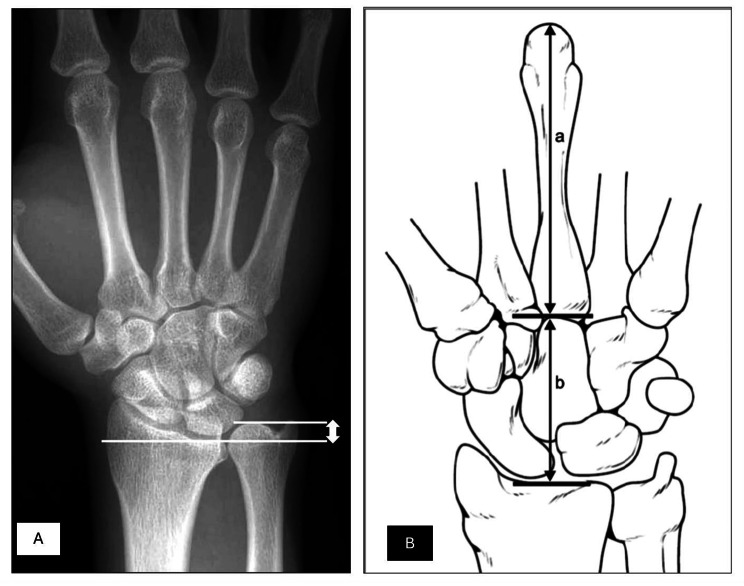
(**A**) Illustration of the radiographic measurement of UV [21]. (**B**) illustration of radiographic measurement of CHR [22].

A total of 385 patients were included in the study, and proper anteroposterior (AP) views of their wrists were used for measurements. Two radiological indices, UV and CHR were calculated. UV was measured in millimeters (mm) as the distance between two tangential lines representing the distal articular surface of the radius and ulna (Fig. 1A). Negative values were recorded for patients with negative UV, while positive values were recorded for patients with positive UV. The CHR was calculated using the method of Youm [19]. Carpal height was determined as the distance between the base of the third metacarpal and the point where the metacarpal axis intersects with the radiocarpal joint [20]. This resulting value was then divided by the length of the third metacarpal (Fig. 1B**).**

### Reliability assessment

In order to assess intra-observer reliability, the hand surgery consultant reader reevaluated the X-rays of 75 randomly selected patients after a minimum interval of four weeks between readings. To evaluate inter-observer reliability, a second independent hand surgeon reader performed identical index measurements on the same set of randomly selected images.

### Statistical analysis

IBM SPSS 22 was used to perform statistical analysis for this study. Descriptive statistics including mean, standard deviation and range were recorded for patients’ demographics (age, gender), and measured radiological indices. A linear regression model with Pearson’s correlation co-efficient was used to study the relation between CHR and UV, and age of the patient and all measured indices. Independent sample t test was used to compare radiological indices between different genders. For reliability analysis ICC was used to measure inter and intra-observer reliability. A significant level was set at *p* < 0.05.

## Results

The demographic characteristics of the study cohort are summarized in the table below, Table 1. The mean age was 41.5 years with a standard deviation of 14.6 years. The gender distribution included 247 males (64.1%) and 138 females (35.9%). Regarding the symptomatic side, 183 patients (47.5%) had right wrist issues, and 202 patients (52.2%) had left wrist issues.

**Table 1:** Characteristics of the study cohorts

**Comparison**		**Mean**	**S.D.**
Age (yrs.)		41.5	14.6
**Comparison**		**No.**	**%**
Gender	Male	247	64.1%
	Female	138	35.9%
Side	Right	183	47.5%
	Left	202	52.2%

### Radiographic measurement

Among the study cohort, the radiological measurement of the CHR, using the Youm method, had a mean value of 0.5 with a range of 0.4 to 1.5. The measurement of UV showed a range from − 5.8 mm (negative ulnar variance) to 4.1 mm (positive ulnar variance), with a mean value of -0.3 mm, Table 2.

**Table 2:** Radiographic measurements (CHR and UV) of the study cohort. In the evaluation of the relationship between age and measured radiological indices, the CHR exhibited a weak negative correlation with increasing patient age, which was statistically significant. Conversely, UV demonstrated a weak positive correlation with patient age, which was not statistically significant, as indicated in table 3

Indices	Mean	SD	Range
Carpal height ratio	0.5	0.06	0.4–1.5
Ulnar variance (mm)	-0.3	2.0	-5.8-4.1

**Table 3:** Correlation between age and radiological indices (CHR and UV), with statistically significant values denoted by an asterisk (*). Comparison of radiographic measures between males and females did not reveal any statistically significant differences in CHR and UV between the two groups, as shown in table 4

Indices	Pearsons’s correlation coefficient (*R*)	*P* value
Carpal height ratio	-0.13	0.03*
Ulnar variance (mm)	0.07	0.2

**Table 4:** Gender-related comparison of the radiographic measures (CHR and UV)

Indices	Male	Female	*P* value
Carpal height ratio	0.53	0.52	0.3
Ulnar variance (mm)	-0.31	-0.38	0.4

**Fig. 2:**
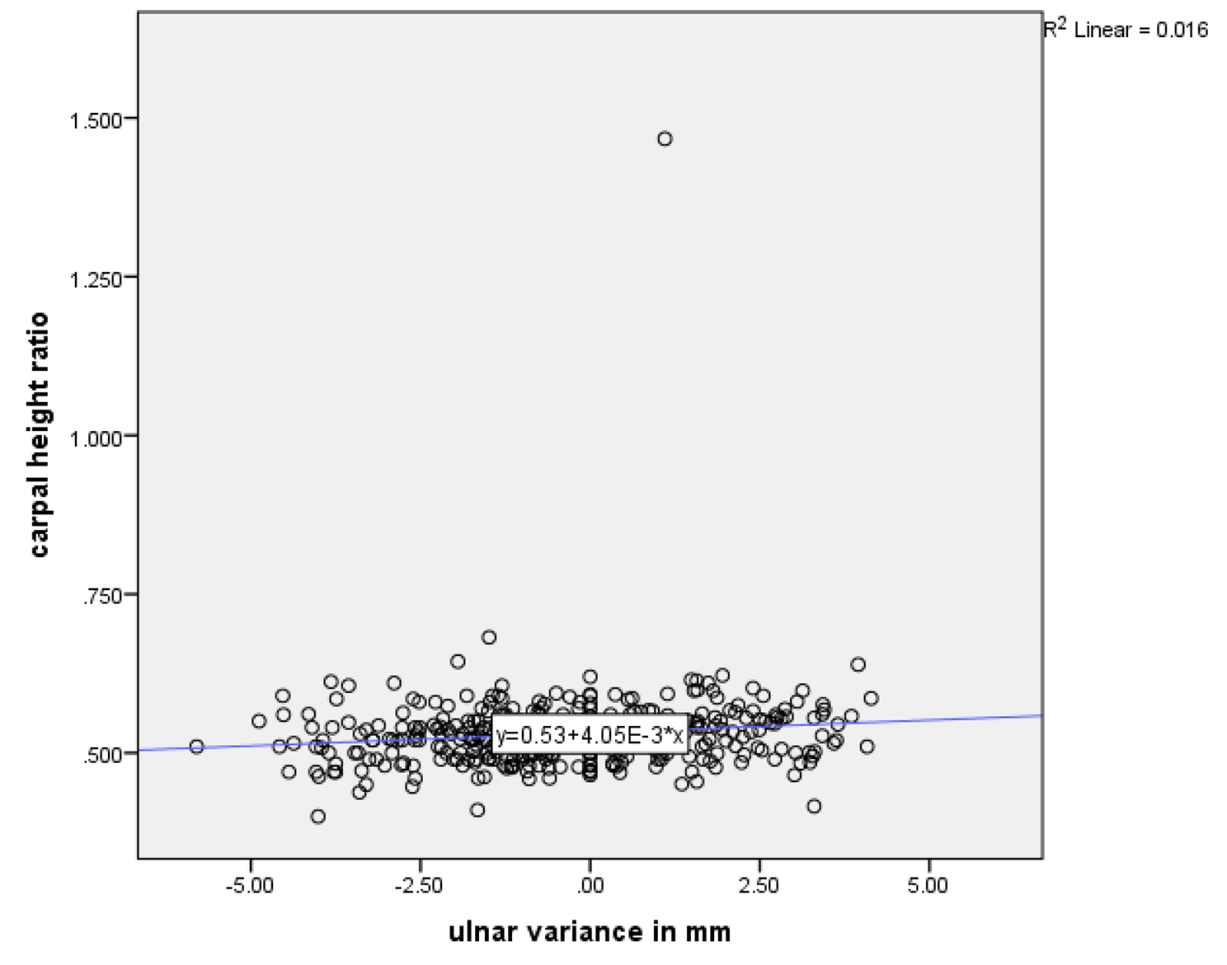
Linear regression model between CHR and UV

An assessment was conducted to investigate the relationship between CHR and UV using a linear regression model and Pearson’s correlation coefficient. It was found that there is a weak positive correlation between the two indices, which was statistically significant (Pearson correlation coefficient of 0.13, *p* = 0.01; adjusted R2 = 0.014, *p* = 0.02 for the linear regression model), Fig. 2.

### Reliability of radiographic measurements

In our analysis of x-ray measurement reliability, we adhered to the guidelines for selecting the intraclass correlation coefficient (ICC) as reported by Koo et al. [23]. A randomly selected x-ray from 75 patients (two views) was used for reliability assessment.

For intra-observer reliability, we employed the ICC two-way mixed effect model with absolute agreement. The results showed high reliability for ulnar variance (ICC = 0.95) and carpal height ratio (ICC = 0.90).

**Fig. 3:**
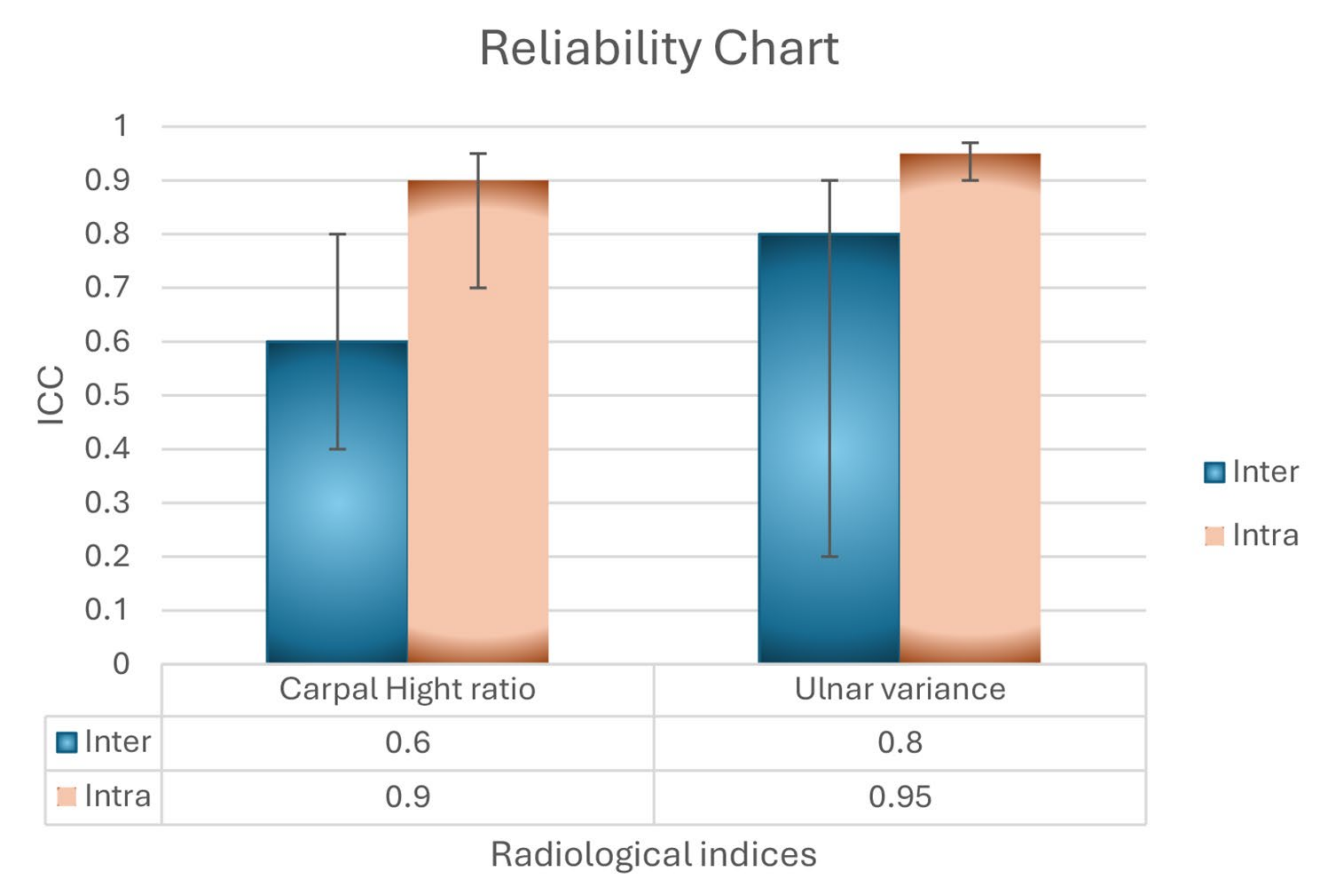
Inter-observer and intra-observer reliability of reading the radiographic measures

Similarly, for inter-observer reliability, we utilized the ICC two-way mixed effect model with absolute agreement. Consistent with the findings for intra-observer reliability, ulnar variance demonstrated good reliability (ICC = 0.80), while carpal height ratio exhibited moderate reliability (ICC = 0.60). These results are illustrated in Fig. 3.

## Discussion

In our study, radiological measurements indicated that age was associated with a weak decline in CHR and a slight increase in UV. The decline in CHR with increasing age was statistically significant. Gender showed no significant association with changes in CHR or UV. A weak positive correlation was also observed between CHR and UV, and this association was statistically significant.

Previous studies have investigated and quantified CHR. In a study involving 120 adult patients, Schuind et al. reported an average carpal height ratio of 0.53 [24]. Additionally, Wang et al. identified a significant difference in CHR between males and females within a Taiwanese cohort (0.52 for men and 0.50 for women aged 20 to 50 years), suggesting the necessity for gender-specific standards [25]. Conversely, Jehan et al. observed no substantial variance in CHR between genders within a Kashmiri population with mean of 0.52, noting no significant divergence between male and female wrists and no notable alterations with age [19]. In our investigation, CHR measurements exhibited minimal differentiation between males and females (0.53 vs. 0.52) yet displayed a statistically significant decline (-0.13) with increasing age.

Previous literature has investigated differences in UV. Sayıt et al., in their analysis of 600 normal wrist X-rays, found disparities between males and females [26]. Nakamura et al. reported a positive correlation between ulnar variance and age in normal wrists, with lower values observed in males compared to females [27]. Conversely, Freedman et al. noticed no significant correlations between ulnar variance measurements and patient age, gender, race, or handedness [28]. Additionally, both Goldfarb et al. and Hollevoet et al. identified variations in ulnar variance based on gender and age group [29, 30]. Our study examined the relationship between ulnar variance and various factors. We observed a weak positive correlation between ulnar variance and increasing age, though this relationship did not reach statistical significance. Additionally, we found no discernible effects of gender on ulnar variance. These results underscore the relevance of age-related considerations in assessing ulnar variance, aligning with previous research highlighting age as a factor influencing these measurements. While our study did not establish a statistically significant association between age and ulnar variance, it emphasizes the importance of accounting for age-related changes in this anatomical parameter assessment.

To the best of the authors’ knowledge of the currently available literature, no previous studies have investigated the relationship between ulnar variance and carpal height ration. In our study, we observed a positive yet weak correlation. Specifically, as ulnar variance increased, there was a corresponding increase in carpal height.

The positive association observed between increased ulnar variance of the wrist and corresponding increase in carpal height ratio in our study may be attributed to several pathoanatomical factors. Ulnar variance, defined as the relative length difference between the ulna and the radius, plays a crucial role in wrist biomechanics, particularly in load transmission across the wrist joint. An increase in ulnar variance can lead to alterations in the distribution of forces within the wrist, potentially influencing carpal height.

One conceivable rationale for this observation pertains to the treatment algorithm proposed by Lichtman et al. for Kienböck’s disease [31]. Current approaches advocate the unloading procedure for the lunate, particularly in the early stages before lunate collapse, when conservative treatments prove ineffective. These procedures aim to redistribute the load on the lunate articular surfaces across adjacent joints, thereby indirectly restoring vascular supply to the lunate bone [32]. To the best of the authors’ knowledge, there is a notable gap in the existing literature regarding the relationship between carpal height ratio and the association of Kienböck’s disease with neutral or positive ulnar variance. This presents an opportune starting point for future research endeavors.

Moreover, the weak positive correlation observed between UV and CHR may be explained by the biomechanical effect of increased ulnar variance, which is known to reduce pressure across the carpal bones [33], This reduction in pressure could potentially reduce the risk of developing conditions such as Kienböck’s disease. Our findings also suggest that advancing age, despite the weak association with increased UV, may contribute to this observed decrease in Kienböck’s disease incidence with age [34]. Additionally, increased UV may affect wrist ligaments and soft tissues, potentially influencing CHR through mechanisms such as ligament laxity or joint instability. It’s important to note, however, that while these correlations were statistically significant, their clinical significance may be limited. Further research is warranted on the potential clinical implications of these weak correlations, particularly in terms of their relevance to Kienböck’s disease. Additionally, elucidating the precise biomechanical pathways underlying the association is crucial for future research efforts, offering valuable insights into wrist condition pathophysiology and informing clinical management strategies.

While our study yielded significant findings regarding the measured radiographic parameters of CHR and UV, we acknowledge certain limitations. Firstly, the measurements were conducted by orthopedic specialists rather than musculoskeletal radiologists for practicality reasons. Additionally, we did not investigate potential factors beyond age and gender that might impact UV and CHR, such as hand dominance, dexterity, or occupational activities. However, it’s important to note that our study primarily focused on age and gender, but these limitations underscore the need for future research to address these factors comprehensively.

## Conclusion

In our study, age was associated with a statistically significant slight decline in carpal height ratio. Gender showed no association with changes in carpal height ratio and ulnar variance. Additionally, we observed a statistically significant yet weak positive correlation between ulnar variance and carpal height ratio.

### Statements

## Data Availability

The data that support the findings of this study are available upon appropriate request from the corresponding author. The data are not publicly available due to privacy or ethical restrictions.

## Abbreviations

CHR: Carpal Height Ratio
UV: Ulnar Variance
